# Primary Cilia, Hypoxia, and Liver Dysfunction: A New Perspective on Biliary Atresia

**DOI:** 10.3390/cells14080596

**Published:** 2025-04-15

**Authors:** Patrícia Quelhas, Diogo Morgado, Jorge dos Santos

**Affiliations:** RISE-Health, Department of Medical Sciences, Faculty of Health Sciences, University of Beira Interior, Av. Infante D. Henrique, 6200-506 Covilhã, Portugal; patricia.quelhas@fcsaude.ubi.pt (P.Q.); a46518@fcsaude.ubi.pt (D.M.)

**Keywords:** ciliopathies, ischemic cholangiopathy, therapeutic interventions, HIF1alpha, biliary atresia, liver

## Abstract

Ciliopathies are disorders that affect primary or secondary cellular cilia or structures associated with ciliary function. Primary cilia (PC) are essential for metabolic regulation and embryonic development, and pathogenic variants in cilia-related genes are linked to several pediatric conditions, including renal-hepatic diseases and congenital defects. Biliary atresia (BA) is a progressive infantile cholangiopathy and the leading cause of pediatric liver transplantation. Although the exact etiology of BA remains unclear, evidence suggests a multifactorial pathogenesis influenced by both genetic and environmental factors. Patients with BA and laterality defects exhibit genetic variants associated with ciliopathies. Interestingly, even isolated BA without extrahepatic anomalies presents morphological and functional ciliary abnormalities, suggesting that environmental triggers may disrupt the ciliary function. Among these factors, hypoxia has emerged as a potential modulator of this dysfunction. Hypoxia-inducible factor 1-alpha (HIF-1α) plays a central role in hepatic responses to oxygen deprivation, influencing bile duct remodeling and fibrosis, which are key processes in BA progression. This review explores the crosstalk between hypoxia and hepatic ciliopathies, with a focus on BA. It discusses the molecular mechanisms through which hypoxia may drive disease progression and examines the therapeutic potential of targeting hypoxia-related pathways. Understanding how oxygen deprivation influences ciliary function may open new avenues for treating biliary ciliopathies and improving patient outcomes.

## 1. Hypoxia and Ciliopathy: Key Players in the Pathogenesis of Biliary Atresia?

Biliary atresia (BA) is a neonatal disease characterized by extrahepatic bile duct obstruction and progressive sclerosing intrahepatic cholangiopathy that frequently leads to chronic liver failure and necessitating liver transplantation (LTx) [1,2,3]. BA is a heterogeneous disease with distinct clinical subtypes, including isolated (non-syndromic) and syndromic forms. The latter is associated with congenital anomalies, such as biliary atresia splenic malformation (BASM) and cardiac-associated biliary atresia (CABA) [4,5,6]. Despite extensive research, the etiology of BA remains unclear, with causal hypotheses ranging from viral infections and immune dysregulation to genetic predisposition and vascular abnormalities [7,8,9,10,11]. One of the main histopathological features of BA is ductular reaction (DR), a reparative process characterized by biliary proliferation, activation of mesenchymal cells and fibrogenesis [12,13,14]. Recent studies have suggested that hypoxia may play a crucial role in modulating this process, and that HIF signaling contributes to both vascular remodeling and cholangiocyte dysfunction [15,16]. Understanding these mechanisms is essential for identifying potential therapeutic targets beyond surgical interventions [17,18].

Previous studies by our group have demonstrated, specifically in the isolated clinical form of BA, a medial layer thickening of hepatic arterial branches [19], immunohistochemical features of vascular endothelial growth factor suggestive of arterial/arteriolar and cholangiocyte hypoxia [15,19]. Overexpression of angiopoietins and their receptors is involved in pericyte recruitment to the arterial wall [20], and a gene expression pattern of hypoxia-ischemia in the liver [10]. Based on these findings, a group in Germany developed a murine model of BA with RVR infection to further investigate the role of vascular disruption and hypoxia in disease progression. Their study revealed a disturbance in the peribiliary vascular plexus (PVP) preceding the biliary luminal obstruction [15,21].

Hypoxia-inducible factors (HIF) 1, 2, and 3 play crucial roles in mediating cellular responses to hypoxia, and their stabilization is associated with primary cilium regression [22]. While HIF-1α and HIF-2α share structural and functional similarities, inducing distinct cellular responses upon binding to the hypoxia-responsive element (HRE) in target genes, HIF-3α acts as a negative regulator of HIF signaling under certain conditions [23]. This process occurs through the activation of HEF1/Cas-L/NEDD9 and Aurora kinase A, which promote histone deacetylase-dependent tubulin depolymerization of the ciliary axoneme [22]. The degradation of HIF-α subunits depends on the von Hippel-Lindau tumor suppressor protein (pVHL), a negative regulator that drives the oxygen-dependent ubiquitin-mediated degradation of these subunits [23]. However, pVHL inactivation prevents HIF degradation, leading to its stabilization and consequent activation of the pathways responsible for ciliary regression. Although the α subunits of HIF-1 and HIF-2 share structural and functional similarities, they induce distinct cellular responses upon binding to the hypoxia-responsive element (HRE) in target genes [24]. These events highlight the intricate role of HIF stabilization in driving ciliopathy-associated processes under hypoxic conditions.

Ciliary abnormalities are frequently observed in BA, although only patients with the syndromic type present with pathogenic variants associated with cholangiociliopathies [5]. In non-syndromic BA, ciliopathy likely represents an acquired disruptive condition, as has been observed in animal models of BA induced by both viruses and toxins [25,26,27]. Primary cilia (PC), membrane organelles present in cholangiocytes, regulate bile secretion in response to bile flow and composition and play a developmental role in biliary patterning, housing multiple signaling pathways [28,29].

### 1.1. Molecular Mechanisms of Hypoxia-Inducible Factors in Ciliary Dysfunction

HIFs are mainly stabilized in response to decreased oxygen availability. Stabilized HIF induces the expression of target genes that maintain biological homeostasis. The levels of HIF-1α and HIF-2α are downregulated in normoxic cells but significantly increase during hypoxia [30], at which point they translocate to the nucleus to induce the transcription of hypoxia-inducible genes [31]. HIF-1α activates more than 70 genes with cell-type-specific responses that include angiogenesis (e.g., increased VEGFA expression), erythropoiesis, glycolysis, vasodilation, and anaerobic metabolism [32,33,34]. Under prolonged or severe hypoxia, HIF-1α can also initiate the transcription of genes involved in cell death pathways [35].

HIF-2α, unlike HIF-1α, is not hydroxylated under hypoxic conditions, preventing its binding to the tumor suppressor pVHL and the subsequent proteasomal degradation. This stabilization allows HIF-2α to promote the transcription of genes associated with erythropoietin synthesis, angiogenesis, cell proliferation and tumor growth [36]. The immunohistochemical detection of nuclear HIF positivity serves as an indicator of HIF pathway activation. Recent findings have linked pVHL, which facilitates the nuclear translocation of HIF-1α, to the maintenance of ciliary integrity in cystic kidney disease [37].

In contrast to HIF-1α and HIF-2α, the role of hypoxia-inducible factor 3 alpha (HIF-3α) remains less understood, although evidence suggests that one of the alternative isoforms may bind to HIF-1α, thereby inhibiting its transcriptional activity [38]. This indicates a potential regulatory role for HIF-3α in modulating the broader hypoxic response, acting as a negative feedback mechanism that limits HIF-1α-driven gene expression under specific conditions.

HIF stabilization has been linked to the presence of a decreased number of cilia, as both processes share common regulatory pathways [39]. In BA, HIF-1α activation is evident in cholangiocytes; however, its precise role in ciliopathy affecting these cells remains unclear [15,16]. While an association between HIF-1α and cilia absence was found, it did not correlate with prognosis [16]. However, cilia counting in BA may be affected by two putative biases: first, quantitative analyses of the presence of cilia are affected by the fact that all stem cells are ciliated, and BA pathophysiology involves the expansion of the progenitor cell niche; second, the proliferation of stem-like cells can be linked to HIF activation. Additionally, although hypoxia, ciliopathy, and the Hedgehog (Hh) signaling pathway have been correlated in other organs, their specific interactions in BA require further investigation [40,41,42]. Specifically, pharmacological inhibition, knockdown, or genetic ablation of HIF-1α abolishes Hh pathway activation [43], which is critical for ciliogenesis and cellular differentiation. Although these mechanisms have been extensively analyzed in other tissues, their implications in hepatic ciliopathy, particularly BA, remain unexplored and warrant further investigation.

### 1.2. Structural Basis of Ciliogenesis

Ciliogenesis is the process by which cilia are formed. It begins with the migration of the basal body to the apical surface of the cell during terminal differentiation [44]. PC develop when a cell exits the cell cycle and enters quiescence, a state in which the cell ceases to divide and becomes metabolically inactive, focusing instead on maintaining homeostasis and performing specialized functions [45].

**Figure 1:**
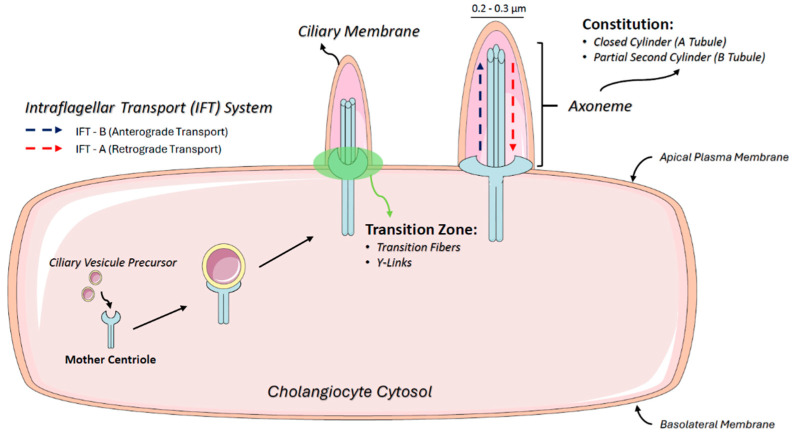
Ciliogenesis and intraflagellar transport mechanisms in cholangiocytes. The schematic illustrates the structure of primary cilia (PC) in cholangiocytes, highlighting their microtubule-based axoneme composed of an A-tubule (closed cylinder) and a B-tubule (partial cylinder). The figure also depicts the basal body derived from the mother centriole, which anchors the cilium to the cytoplasm. Additionally, intraflagellar transport (IFT) mechanisms are shown, including anterograde transport via IFT-B and retrograde transport via IFT-A, essential for ciliary assembly, maintenance, and signaling.

PC has a diameter of 0.2–0.3 microns and lacks compartments such as the endoplasmic reticulum (ER) and free ribosomes. Assembly and maintenance involve the intraflagellar transport (IFT) system, consisting of IFT complex A (IFT-A) for retrograde transport, IFT complex B (IFT-B) for anterograde transport, and BBSome, a protein complex that operates in PC biogenesis and homeostasis and is involved in transporting signaling molecules inside and outside PC [44] (Figure 1).

PC are structured by microtubules composed of alpha- and beta-tubulin dimers that form a closed cylinder (A-tubule) coupled with a partial second cylinder (B-tubule), collectively referred to as an axoneme. At the basal end, the doublet microtubules extend directly from the centriole microtubules, anchoring PC to the cytoplasm. The basal body, which is derived from the mother centriole, contains nine symmetrical microtubule triplets. Distal appendages extend outward from the basal body, docking it to the plasma membrane and facilitating ciliogenesis. The transition zone, located between the distal end of the proximal region of the axoneme and the basal body, includes transition fibers and Y-links that connect microtubule doublets to the ciliary membrane [46].

To concentrate and regulate signaling molecules within the ciliary membrane, membrane cargo is transported in vesicles from the cytoplasm to the cilium base. The lateral movement of these molecules into and out of the ciliary membrane compartment is restricted by a diffusion barrier at the basal portion of the cilium, primarily formed by the transition zone [47], which is critical for maintaining the distinct composition of the ciliary membrane, an essential structure for signaling function [44].

### 1.3. Primary Cilia Play a Critical Role and Function in the Liver

PC are highly conserved organelles found on the surfaces of most growth-arrested or differentiated mammalian cells. They play crucial roles in regulating various cellular processes and maintaining cellular homeostasis [48], such as cell proliferation, differentiation, migration, cell polarity, signaling pathways, and other vital activities.

In the liver, PC are present in cholangiocytes and hepatic progenitor cells but are absent in mature hepatocytes, as progenitor cells lose their cilia upon differentiation [49]. Injured biliary epithelium regenerates from ciliated progenitor cells, and intrahepatic bile ducts rarely undergo hepatocyte metaplasia, although this remains debatable [13,50]. In extrahepatic ducts, regeneration relies on biliary cell proliferation in the peribiliary glands [51]. Under normal conditions, cholangiocytes maintain homeostasis through self-replication, losing their PC during mitosis, and restoring them upon maturation [52]. This cyclical reconstruction of PC aligns with cholangiocyte differentiation. While liver repair is often termed “regeneration”, post-resection recovery in mammals is better described as “compensatory hyperplasia”, as it expands functional capacity rather than replacing lost lobes.

**Figure 2:**
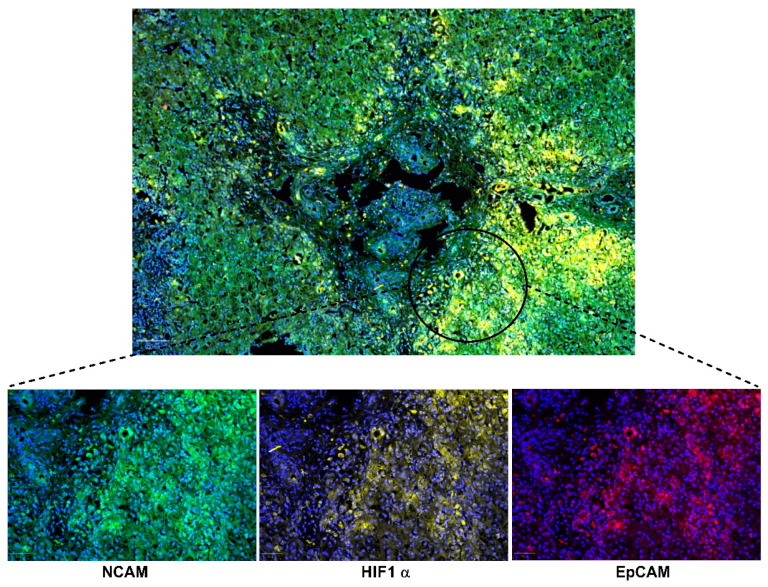
Representation of the cellular niche in biliary atresia, including mesenchymal cells, inflammatory cells, and myofibroblasts, which contribute to ductular reaction-associated fibrogenesis. Images were obtained from formalin-fixed paraffin-embedded liver tissues of children with Biliary Atresia. Staining highlights different markers: DAPI (nuclei, blue), HIF-1α (yellow, hypoxia marker), NCAM (Neural Cell Adhesion Molecule, associated with hepatic progenitor or mesenchymal cells, green), and EpCAM (Epithelial Cell Adhesion Molecule, marking epithelial cells, including cholangiocytes, red). Magnification: 20×.

From a histopathological perspective, BA displays DR and precocious fibrogenesis [53,54]. In BA, DR arises from the proliferation of progenitor cells located in the Hering canal within the Space of Mall [55] in the periportal region. These progenitor cells, including hepatoblasts, have the potential to differentiate into biliary and hepatocytic lineages, contributing to ductular reactions and liver regeneration. This niche also includes mesenchymal cells, inflammatory cells, and myofibroblasts, which contribute to the fibrogenesis associated with the ductular reaction (Figure 2) [56,57]. Additionally, some biliary structures in BA resemble the “ductal plate malformation” (DPM) characteristic of cholangiociliopathies [13]. Although hepatocytic metaplasia at the portal interface has been suggested [58], its biological significance remains unclear.

Progenitor cells are involved in replacing lost liver parenchyma [59]. Specifically, the terminal segment of the biliary system, known as the canal of Hering, harbors progenitor cells capable of generating both hepatocytes and cholangiocytes under specific conditions. Lineage tracing experiments in rats and zebrafish have demonstrated hepatocyte regeneration from biliary stem cells [60]. However, in mice, the cre/lox model did not show biliary-derived hepatocytes, although it confirmed the hepatocytic origin of biliary cells in cholangiocarcinomas [61]. Subsequent studies revealed that hepatic progenitor cells of biliary origin have liver repopulation capacity in mice when hepatocyte proliferation is blocked [62].

## 2. Liver Function and Ciliopathies

In the liver, cholangiocytes, Kupffer cells, and endothelial cells each have cilia that play distinct and essential roles in maintaining liver health. Primary cilia in cholangiocytes act as mechanosensors and chemosensors, allowing them to detect changes in bile flow, composition, and osmolality [29,63,64,65]. These cilia integrate various signaling pathways, including Hedgehog, Wnt, and Notch, which are crucial for biliary development, repair, and homeostasis [66,67,68,69]. Dysfunction of these cilia can disrupt the regulation of bile flow and contribute to pathological conditions.

One important mechanism regulated by cholangiocyte cilia is calcium signaling [65]. Ciliary proteins, such as polycystin-1 and polycystin-2, form a complex within the primary cilium that facilitates the entry of extracellular calcium ions (Ca^2+^), thereby influencing intracellular signaling pathways [65]. Additionally, transient receptor potential cation subfamily V member 4 (TRPV4) channels in cholangiocyte cilia respond to changes in bile osmolality by activation when bile tonicity decreases and inhibition when it increases [70]. This mechanism regulates intracellular Ca^2+^ levels and subsequent cellular responses. Cholangiocyte cilia also possess chemosensory capabilities, with receptors such as P2Y12, which are activated by biliary nucleotides (ATP and ADP), thereby affecting cAMP signaling pathways [71].

Pathogenic variants in genes that encode structural or accessory proteins of PC can lead to disorders known as “ciliopathies” [48]. These conditions can manifest at different stages of life, including embryonic development, childhood, and adulthood. Ciliopathies often affect multiple organs and systems, leading to a wide range of overlapping symptoms.

Defects in PC structure and function lead to ductular reactions and changes in bile fluidity, thereby disrupting bile homeostasis [14]. Intact PC-based signaling is essential for the normal development of portal triads, including the branches of the biliary tree, and disruption of ciliary function can lead to a range of liver disorders.

Most hepatic fibrocystic diseases result from defective ciliary proteins, except autosomal dominant polycystic liver disease (ADPLD) and portal fibrosis associated with congenital disorders of glycosylation (CDG) type Ib [72]. The most common conditions include Congenital Hepatic Fibrosis (CHF), characterized by defective ductal plate remodeling, portal vein abnormalities, arterial hyperplasia, and progressive fibrosis [73,74]; Caroli Disease (CD), marked by macroscopic dilations of medium and large intrahepatic ducts [73]; and Polycystic Liver Disease (PLD), involving isolated cysts derived from biliary microhamartomas (Von Meyenburg Complexes), which are typically unconnected to the intrahepatic biliary tree [75,76].

Numerous studies have underscored the role of primary cilia (PC) in the pathophysiology of liver diseases (see Table 1). Pathogenic variants in genes such as PKD1 and PKD2 (related to Polycystic Liver Disease) [77,78], PKHD1 (associated with Congenital Hepatic Fibrosis and Caroli Syndrome) [79,80], and DCDC2 (linked to Neonatal Sclerosing Cholangitis and Biliary Atresia) [81,82] contribute to bile duct malformations. Pathogenic variants in IFT88, which are related to Bardet-Biedl Syndrome, can lead to liver fibrosis [72], while ALMS1 (linked to Alström Syndrome) [83,84,85] and NEK8 (associated with Nephronophthisis) [86,87,88] are involved in renal cysts and liver fibrosis. Pathogenic variants in ARL13B (related to Joubert Syndrome) and MKS1 (associated with Meckel-Gruber Syndrome) result in hepatic fibrosis and cysts, respectively [89,90,91,92]. Additionally, variants in TMEM67 are associated with liver disease in Joubert Syndrome [93]. Dysregulation of mTORC1 impacts nutrient sensing, autophagy, and stress responses, further exacerbating liver ciliopathies [45,94].

### 2.1. Ciliary Dysfunction in Biliary Atresia: Insights and Implications

#### 2.1.1. Genetic Variants in Ciliary Function and Their Impact on Biliary Atresia

**Figure 3:**
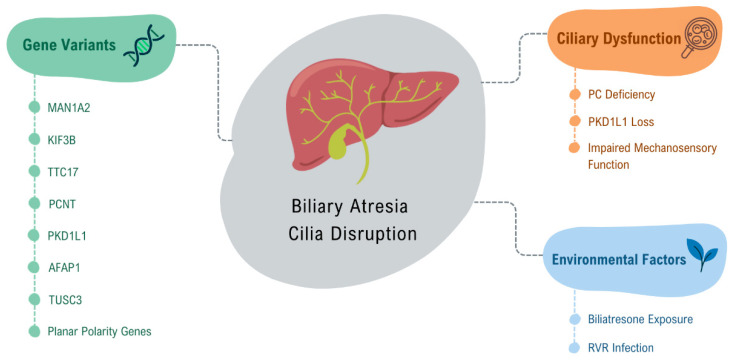
Factors contributing to ciliary disruption in Biliary Atresia. This figure illustrates the key genetic variants associated with ciliary dysfunction in biliary atresia, including MAN1A2, KIF3B, TTC17, PCNT, and PKD1L1, and environmental factors that contribute to BA pathogenesis, such as Biliatresone exposure and Rotavirus Rhesus infection. These factors disrupt ciliary structure and function, contributing to the pathogenesis of BA by impairing biliary flow and cellular signaling.

The key role of primary cilia (PC) in maintaining biliary and hepatocellular health highlights the significance of ciliary function in preserving biliary homeostasis. This suggests that defects in the cilia may contribute to the development and progression of biliary atresia (BA) (Figure 3). A study conducted by Hellen et al. (2023) utilized bile duct ligation in KD1L1-deficient mice, successfully replicating the critical intrahepatic pathophysiological features of BA, including peribiliary fibroinflammation, hepatic arteriopathy, and ciliopathy [99]. This research provides a valuable opportunity to enhance our understanding of the disease and identify potential therapeutic targets [99].

Gene variants in MAN1A2 [96], kinesin-like protein KIF3B (KIF3B), tetratricopeptide repeat domain 17 (TTC17), pericentrin (PCNT) [97], and PKD1L1 [98,100,101] have been identified in patients with BA, contributing to this ciliary dysfunction. Lim et al. (2024) studied variants of the PKD1L1 gene, whose loss mimics syndromic BA in mice, and demonstrated that this loss causes ciliary dysfunction by disrupting ciliary signaling, thus classifying syndromic BA as a cholangiociliopathy [102]. These findings highlight the critical role of cilia and related gene mutations in the pathophysiology of liver diseases, including BA [103].

Glessner et al. (2023), through genome-wide association studies (GWAS) involving 811 biliary atresia patients, indicated that abnormal biliary development in BA is, in part, due to disruption of ciliogenesis and ciliary function through genes such as AFAP1 (actin filament integrity modulator) and TUSC3 (protein involved in cellular magnesium uptake) [104]. Additionally, they showed through an integrative analysis of hepatic BA transcription that downstream disruption of vascular and epithelial tube morphogenesis may explain portal vein atresia, which occurs in some cases of BA, indicating a possible association between BA and vascular development [104]. Karjoo et al. (2013) demonstrated, in a murine model of BA with rhesus rotavirus (RRV) infection, as well as in human tissue, that there is significant loss of extrahepatic cholangiocyte cilia, indicating the possibility that BA is an acquired ciliopathy [27].

A recent study by Hai-Bing et al. (2024), which evaluated the effect of biliatresone on human liver organoids, confirmed that exposure to this toxin causes morphological and functional changes similar to those observed in BA [105]. The organoids exposed to biliatresone exhibited impaired ciliary function in cholangiocytes, characterized by a decreased number of cilia and compromised mechanosensory function [105]. In a previous study by Lorent et al. (2015), the effects of biliatresone on extrahepatic cholangiocytes were evaluated [106]. The researchers observed that cells treated with biliatresone experienced a reduction in primary cilia length, and staining for cellular tubulin revealed a dose-dependent decrease in the number of visible microtubules. This suggested that biliatresone negatively affected microtubule stability [107]. Additionally, spheroids designed to mimic BA were utilized in that study, which led to the conclusion that biliatresone resulted in the loss of epithelial monolayer integrity, lumen closure, and disruption of apical-basal polarity [106].

**Figure 4:**
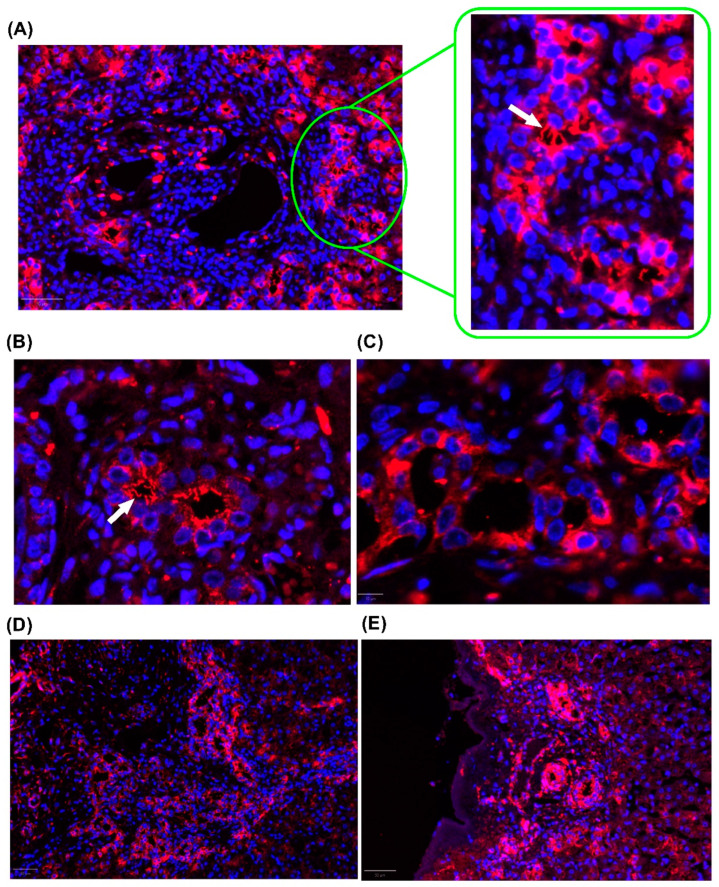
Representative image of cilia in cholangiocytes. Fluorescence microscopy images showing cilia labeled with TUBA4A (red) and nuclei stained with DAPI (blue). (**A**) Cytoplasmic expression and evident cilia in the portal space. White arrows indicate primary cilia. (**B**) Higher magnification (40×) image of the bile duct, highlighting prominent cilia in cholangiocytes. White arrow indicates primary cilia. (**C**) Control bile duct without primary cilia. (**D**) Cytoplasmic staining of cholangiocytes at the interface region. (**E**) Cilia and cytoplasmic staining in the biliary ducts in the subcapsular region. The images shown were obtained from formalin-fixed paraffin-embedded liver tissue from children with Biliary Atresia. Magnification: 20×.

Another line of investigation in ciliopathies in BA was highlighted in a recent study by our group, which showed that reduced PC length, PC bending, and increased cytoplasmic tubulin expression occur in the isolated clinical form of BA. Our findings suggest that a disruption in tubulin transport between the cytoplasm and PC negatively impacts early prognosis after portoenterostomy (Figure 4) [16].

#### 2.1.2. The Role of Ciliary Genes in BASM

Approximately 20% of patients with BA exhibit a syndromic form known as embryonic BA, which includes conditions such as BASM [6,108]. BASM is characterized by defects in laterality determination, which can lead to issues like malrotation, dextrocardia, and polysplenia [25]. These findings suggest that ciliary defects may play a significant role in the pathophysiology of these conditions [25].

During embryogenesis, abnormal function of primary cilia has been linked to laterality defects, highlighting the importance of proper ciliary structure in these developmental processes. Research by Chu et al. (2012) showed that cilia in liver specimens from patients with BA, regardless of laterality defects, were significantly shorter and less abundant than those in normal livers and other neonatal cholestatic diseases [25]. Similarly, Mitra et al. (2021) demonstrated notable abnormalities in primary cilia in patients with BA, with a significant reduction in their number compared to healthy controls [107].

Another study by So et al. (2020) indicated that the necessity for liver transplantation in BA cases may be influenced by sequence variants in the mannosidase alpha class 1A member 2 (MAN1A2) gene [96]. This gene interacts with the ARF6 and EGFR signaling pathways to regulate the development of the intrahepatic biliary network. Furthermore, it was observed that both MAN1A2 mRNA and protein expression levels were lower in the liver tissue of BA patients, potentially due to the gene’s role in proper laterality determination and hepatobiliary morphogenesis through its influence on ciliogenesis [96,109].

A novel hypothesis suggesting that primary cilia (PC) play a role in the pathogenesis of biliary atresia (BA) may lead to further investigations into the genes associated with ciliary development. This research could focus on patients presenting with laterality defects and other forms of BA linked to genetic susceptibility.

## 3. The Role of Hypoxia

### 3.1. Biliary Hypoxia: Mechanisms and Impacts

Investigating the role of hypoxia in hepatic and biliary tissues is becoming increasingly important. de Jong et al. (2022) showed that chronic biliary hypoxia resulting from microvascular disruption can lead to the development of non-anastomotic biliary structures [50]. This biliary hypoxia triggers the proliferation and differentiation of peribiliary gland stem cells into mature cholangiocytes, underscoring the critical importance of oxygen supply to the biliary anatomy and the potential implications of hypoxia in biliary diseases [50].

In humans, both extrahepatic and intrahepatic biliary structures receive their blood supply exclusively from the arteriolar branches of the hepatic artery, which form the peribiliary vascular plexus (PVP). Recent studies on biliary atresia (BA) suggest that disruption of the PVP may initiate biliary hypoxia. In the livers of patients with BA, there is evidence of progressive thickening of the medial layer in hepatic artery branches [110], peripheral arterial blockages accompanied by the formation of perivascular arteriolar tufts [111], and immunohistochemical expression of angiogenic factors within the biliary structures [19]. These findings indicate the presence of hypoxia affecting the biliary tree and the occurrence of reactive angiogenesis.

**Figure 5:**
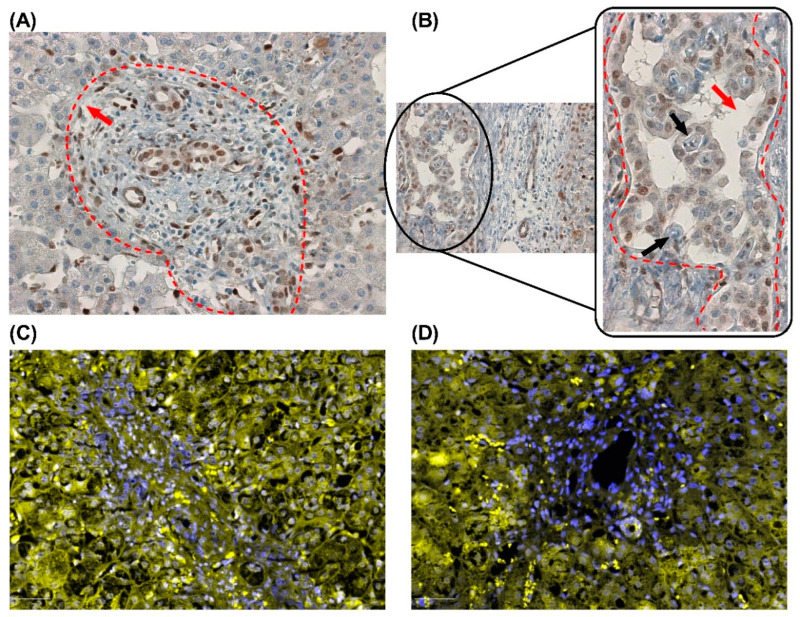
Representative images of nuclear positivity for HIF-1α. (**A**) Immunohistochemistry image of the portal area showing cholangiocyte nuclei strongly positive for HIF-1α (brown staining). HIF+ staining is observed in the portal area, interlobular bile ducts, peribiliary vascular plexus (PVP) endothelium, and interface (Mall’s space). The dashed red line delimits the Portal Space, and the red arrow indicates the Mall Space. (**B**) Immunohistochemistry image with HIF+ staining in a structure resembling a Ductal Plate Malformation (DPM) (left) and at the interface of a fibrovascular septum (upper right). The dashed red line delimits the Space of Mall, with the red arrow indicating the same. Black arrows indicate PVP. (**C**) Immunofluorescence image showing intense nuclear HIF-1α staining associated with the development of a fibrovascular septum. (**D**) Immunofluorescence image of a portal area showing bile ducts with HIF+ cholangiocytes. HIF-1α is labeled yellow and DAPI blue. The images shown were obtained from formalin-fixed paraffin-embedded liver tissue from children with Biliary Atresia. Magnification: 20×.

Our research group demonstrated in 2014 that liver specimens from patients with the isolated form of BA showed upregulation of angiopoietins and their receptors, which are crucial for recruiting pericytes to the vascular wall [20], along with hypoxia-ischemia molecular features linked to disease progression [10]. Additionally, we found that activation of the HIF-1α pathway may play a role in the pathogenesis of BA, potentially involving ischemic cholangiopathy (IC) and/or disruption of the REDOX state (Figure 5) [15].

Ischemic cholangiopathy involves focal or extensive damage to the bile ducts due to reduced blood supply, commonly seen in LTx, hepatic arterial infusions of toxic agents, and certain vascular conditions [112,113,114] that compromise bile duct integrity through ischemia. PVP supplies oxygen to the intrahepatic bile ducts, and obstruction of its small vessels (<200 µm) leads to biliary duct ischemia and fibrotic strictures [10,115]. During procedures such as LTx, cholangiocytes are highly sensitive to short ischemic episodes, resulting in structural alterations that impair ductal secretory function post-reperfusion [116,117,118]. This lack of adequate blood supply can also affect the peribiliary glands, which harbor stem cells essential for biliary epithelium regeneration [119], and large bile ducts, which consist of extrahepatic, segmental (400–800 µm), and area (300–400 µm) ducts [120]. Experimental models have indicated that hypoxia triggers periportal expression of factors such as VEGF and fibroblast growth factor-2 (FGF-2) [121], affecting cholangiocytes and hepatocytes and potentially promoting structural changes linked to cholestasis and biliary dysfunction [112,116].

In addition to the lack of oxygen supply during LTx, the post-transplant reperfusion process is also a critical factor that can lead to LTx failure due to the occurrence of large-scale REDOX stress [122,123]. Ischemia-reperfusion injury affects not only hepatocytes and liver endothelial cells but also damages biliary structures. Biliary complications after LTx occur in 10% to 30% of operated patients and lead to increases in graft dysfunction, patient morbidity, graft loss, retransplantation, and even death rates [116].

### 3.2. Relationship Between Hypoxia and Ciliary Function

#### 3.2.1. Hypoxia and Ciliopathy in Liver Diseases

Hypoxia plays an important role in the pathogenesis of different liver disorders [124], including lesions caused by hepatic ischemia/reperfusion [123,125]. Cholestatic disorders can arise from liver ischemia through various mechanisms. In a rat model of arterial liver ischemia, hypoxia primarily affects cholangiocytes and hepatocytes, predominantly in the periportal area after complete arterial deprivation. In liver tissue, there is a marked reduction in the expression of hepatocyte membrane transporters, even before bile duct proliferation occurs [118]. In the clinical context, postoperative hyperbilirubinemia in patients undergoing liver resection, a procedure that includes vascular clamping and thus hypoxia, is associated with high morbidity and mortality [126]. New studies are increasingly focusing on how hypoxia affects PC of the liver, especially in relation to ischemia-reperfusion and its role in biliary injury, as hypoxia-related damage affects biliary structures in addition to hepatocytes [15].

A study by Esser et al. (2024) showed that liver pre-transplantation ischemic conditions shorten PC in biliary epithelial cells, and that stabilization of PC is an effective method for preventing biliary epithelial cell apoptosis, contributing to transplant success [127]. In that study, it was shown that damage to PC during ischemia triggers cellular senescence in biliary epithelial cells. Consequently, these cells lose their ability to proliferate, which leads to persistent biliary injury and impaired regeneration [127].

To our knowledge, the first description of HIF-1α nuclear positivity in cholangiocytes of the intrahepatic biliary tree was obtained by our group and may be associated with hypoxia, oxidative stress, and molecular pathways involved in ciliary disruption [15].

#### 3.2.2. Ciliary Alterations Driven by Hypoxia Beyond the Liver

##### HIF-1α: A Fundamental Player in Hypoxia and Cilia Dynamics

The impact of hypoxia on cilia has been a focus of research interest, not only in the liver but also in various other organs. Studies have shown that the HIF pathway and its associated genes, such as IFT52 and VHL, play critical roles in regulating ciliogenesis under different oxygen conditions, providing insights into how oxygen availability affects ciliary function in various tissues. Brown et al. (2003) observed in *Tetrahymena thermophila* that the protein IFT52p (encoded by the IFT52 gene) is involved in signaling pathways that regulate cilia assembly, which may be particularly sensitive to hypoxic conditions [128]. The observed suppression of cilia assembly in IFT52 mutant cells suggests a direct link between ciliogenesis and hypoxia-mediated signaling, emphasizing the potential role of hypoxia in modulating ciliary dynamics at a fundamental level [128].

Research on the von Hippel-Lindau (VHL) gene has focused on its role in regulating hypoxia through the HIF-1α pathway. Lutz and Burk (2006) examined how the VHL gene contributes to the formation of primary cilia (PC) in renal-derived cells and its influence on hypoxia via the HIF-1α pathway [129]. VHL is essential for the ubiquitination process, as it facilitates the degradation of HIF-1α under normoxic conditions, thereby regulating the cellular response to hypoxia. Their study found that cells lacking functional VHL failed to develop cilia, leading to disrupted ciliogenesis, which is linked to renal cyst formation and the development of renal cell carcinoma. The significance of VHL in maintaining primary cilia has been emphasized, along with its role in mediating the HIF-1α pathway [129].

Further research by Troilo et al. (2014) highlighted the involvement of ubiquitin-specific protease 8 (USP8) in ciliogenesis, which works in conjunction with the VHL protein pVHL, as demonstrated through siRNA-based screening [130]. They found that USP8 acts as a deubiquitinating enzyme for HIF-1α, counteracting pVHL-mediated ubiquitination of HIF-1α. This interaction maintains basal levels of HIF-1α expression and HIF transcriptional activity under normoxic conditions, which is crucial for endosome trafficking and ciliogenesis [130].

Additional studies have shown interactions between HIF-1α and cellular components related to ciliogenesis in renal carcinoma and other hypoxia-sensitive tissues. For instance, mitotic aurora kinase A (AURKA) has been identified as a specific target of HIF-1α in the VHL syndrome. Dere et al. (2015) suggested that HIF-1α inhibits AURKA through β-catenin-directed transcription [131]. VHL protein (pVHL) regulates HIF subunits under normoxic conditions by promoting ubiquitination and subsequent proteasomal degradation. This process prevents the stabilization and nuclear translocation of HIF-1α and HIF-2α, thereby maintaining cellular homeostasis.

**Figure 6:**
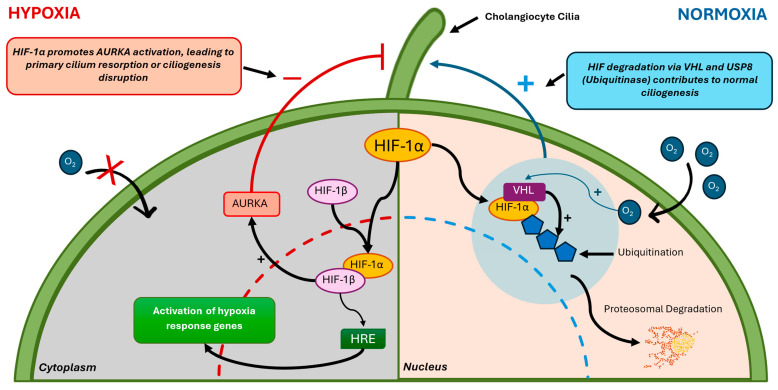
Schematic representation of the relationship between HIF-1α, VHL, AURKA, and primary cilia in hypoxic and normoxic conditions. Normoxia (right side): Under normal oxygen levels, HIF-1α is ubiquitinated by the von Hippel-Lindau protein (VHL) and degraded via the proteasomal pathway. USP8, a deubiquitinating enzyme, counteracts VHL-mediated HIF-1α ubiquitination to maintain the basal HIF transcriptional activity. This regulation contributes to normal ciliogenesis, as VHL plays a crucial role in maintaining primary cilia integrity. Hypoxia (left side): Under low-oxygen conditions, HIF-1α is stabilized and translocates to the nucleus, where it dimerizes with HIF-1β and binds to hypoxia-responsive elements (HREs) to activate the transcription of hypoxia-related genes. Hypoxia also promotes the activation of AURKA, leading to primary cilium resorption or disrupted ciliogenesis.

Recent findings also underscore the role of pVHL in maintaining the integrity of primary cilia, particularly in the context of cystic kidney disease. Dysfunction of pVHL disrupts ciliogenesis and endosome trafficking, leading to the stabilization of both HIF-1α and HIF-2α, resulting in the upregulation of AURKA (Figure 6) [132].

Research has also explored the molecular mechanisms by which hypoxia influences ciliogenesis. Fabbri et al. (2020) examined the regulation of ciliogenesis in renal carcinoma cells, with a focus on the hypoxic form of the voltage-gated anion channel (VDAC1-ΔC) [133]. Their findings indicate that PC loss in these cells is dependent on the pVHL/HIF/hypoxia pathway [133], supporting the idea that changes in cellular structure and function due to hypoxia are associated with VHL/HIF signaling.

Furthermore, HIF-1α appears to affect ciliary architecture and motility in tissues other than the kidneys. Resnick (2016) discovered that cilia in tissues stabilized by HIF were more flexible than those in non-stabilized tissues [39]. This flexibility may suggest that cells adjust cilium length and flexibility in response to fluctuating oxygen levels [39]. Their study indicated that longer cilia are associated with increased flexibility, implying that cells regulate cilium length to maintain consistent sensitivity and functionality under varying oxygen conditions [39].

In primary human nasal epithelial cells, HIF-1α has been linked to cilia loss and increased proliferation of goblet cells, potentially through the phosphorylation of NLRP3, an essential component of the inflammasome [134]. Similarly, HIF-1α regulates primary cilia formation in HeLa cells via ROS-induced NPHP3 expression and ERK activation during serum deprivation, highlighting its role in adapting to stress [135]. In mesenchymal stem cells (MSC), silencing HIF-1α prevents cilia loss under hypoxic conditions, while constitutively active HIF-1α reduces primary cilia formation, underscoring its critical role in maintaining ciliary stability under low-oxygen conditions [37]. A recent study from our group examined the effects of hypoxia and HIF-1α pathway activation on primary cilia in cholangiocytes and found an inverse relationship between HIF-1α expression and the presence of cilia. This suggests that hypoxia disrupts ciliary formation and maintenance, contributing to biliary dysfunction [16].

##### Impact of HIF-2α on Primary Cilia

Recent findings have suggested that HIF-2α plays a role in PC signaling beyond its transcriptional activity. Specifically, HIF-2α appears to contribute to the pathology of osteoarthritis by promoting PC loss through the HIF-2α/AURKA/NEDD9 pathway, whereas HIF-1α does not exhibit this effect [42]. Leu et al. (2023) further identified the accumulation of HIF-2α in the ciliary axoneme, where it interacts with IFT88, influencing MEK/ERK signaling, and enhancing cell survival under hypoxic conditions [136]. Similarly, Johnston et al. (2024) demonstrated that increased HIF-2α activity upregulates PC-related genes and promotes longer cilia in hypoxic murine neuronal cells through interactions with an IFT88 homolog, potentially aiding cellular adaptation to low-oxygen levels [137].

To examine the relationship between primary cilia and HIFs in inflammatory signaling, Wann et al. (2013) conducted a study using primary articular chondrocytes from both bovine and human sources [138]. They concluded that the sequestration of HIF-2α in cilia negatively regulates its expression and may influence HIF-2α activity, illustrating for the first time that primary cilia play a regulatory role in HIF signaling during inflammation [138]. Qiao et al. (2021) investigated the roles of HIF-1α and HIF-2α in the PC of human retinal epithelial cells and found that hypoxia elongated cilia in a dose-dependent manner when using CoCl_2_ (a model for hypoxia), with this increase being reversible and dependent on exposure time [139]. This finding contrasts with observations in the liver, where hypoxia appears to impact ciliary structure and function differently [127]. This suggests that hypoxia-induced ciliary elongation serves as a cellular adaptation mechanism, albeit with varying modulation across different tissues.

##### Hypoxia and mTOR: Effects on Ciliary Function and Autophagy

**Figure 7:**
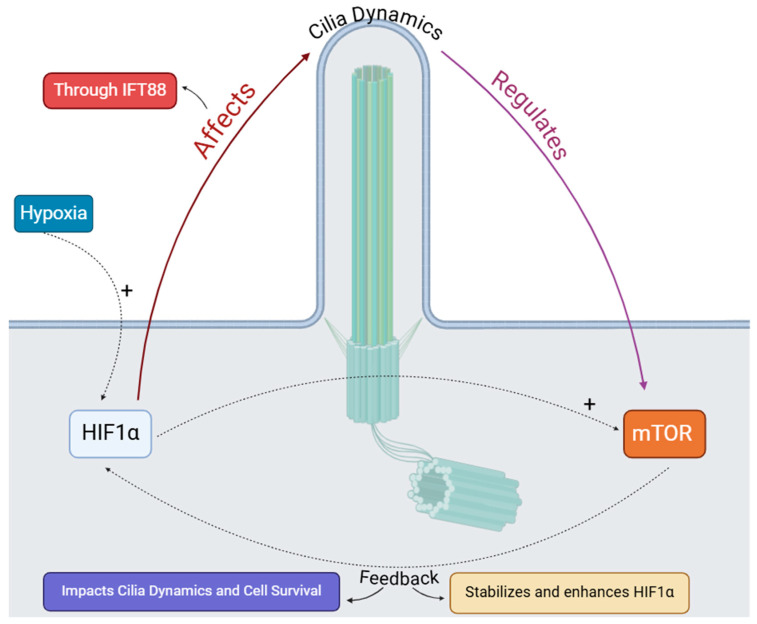
Interaction between hypoxia, mTOR pathway, and cilia dynamics. Hypoxia activates HIF1α, which influences ciliary function and cell survival. The mTOR pathway plays a stabilizing role by enhancing HIF1α activity in response to cellular stressors. Arrows indicate regulatory interactions, with feedback mechanisms modulating the overall dynamics of the cilia and cellular adaptation.

Another molecular pathway affected by hypoxia is the mTOR pathway, which is a member of the PI3K-related kinase family. This pathway consists of two distinct complexes: mTORC1 and mTORC2, both sensitive to rapamycin [140,141]. Each complex has unique functions and associated proteins that account for its different roles. mTORC1 primarily regulates cell growth and metabolism, while mTORC2 is involved in cell survival and cytoskeletal organization. Hypoxia can disrupt both pathways, leading to various cellular responses [95]. Wang et al. (2015) demonstrated that cilia and autophagy influence each other reciprocally, suggesting that the mTOR pathway is integral to this regulation [142]. Specifically, suppression of mTOR signaling enhances autophagy, promoting ciliary elongation, while increased mTOR activity shortens cilia by inhibiting autophagic processes (Figure 7). These findings emphasize the complex relationship between cellular pathways and ciliary dynamics, indicating that mTOR plays a key role in the interaction between autophagy and ciliogenesis [142]. Recent research supports this complex relationship. Morleo et al. (2023) proposed that primary cilia serve as specialized sites for the control of autophagy, with selective autophagic degradation impacting both cilia formation and elongation [143]. However, the impact of autophagy on ciliogenesis appears to be context-dependent, as contradictory findings have been reported [143]. The complexity of this interaction is further underscored by emerging evidence implicating mitochondria and lysosomes in ciliary regulation, although their precise roles remain unclear [144,145,146].

The increasing number of genes associated with both ciliopathies and autophagy regulation highlights the broader implications of this interaction in diseases [147]. As suggested by Morleo et al., targeting autophagy could offer therapeutic potential for ciliopathies, and ciliogenesis itself might serve as a useful biomarker for therapies aimed at modulating autophagy in diseases that extend beyond ciliary dysfunction [143].

Evidence indicates a connection between the mTOR and HIF-1α pathways. Sakamoto et al. (2014) demonstrated that crosstalk between MT1-MMP and mTOR inhibits HIF-1, which leads to the activation of HIF-1α [148]. The activation of the PI3K/mTOR pathway increases the levels of the HIF-1α protein by enhancing its translation, rather than affecting mRNA expression [149]. Furthermore, overexpression of Rheb activates mTOR, which boosts HIF transcriptional activity under hypoxic conditions; this effect can be reversed by rapamycin [150,151,152]. Additionally, mTOR regulates HIF-1α through CAP-dependent translation mechanisms, which influence its stabilization and synthesis [150].

## 4. Potential Therapeutic Interventions

With increasing evidence highlighting the role of ciliopathies in liver diseases, therapeutic strategies focused on preserving primary cilium integrity, especially regarding biliary health, could greatly improve native liver survival and enhance outcomes of liver transplantation. This area presents excellent opportunities for further research. Potential strategies include the use of senolytics and treatments that stabilize cilia to maintain their structure and function during and after liver transplantation [127,153].

Defects in ciliary structure or function lead to decreased intracellular calcium and increased cAMP levels, making these pathways promising therapeutic targets [65]. In a PCK rat model of polycystic kidney and liver disease, Masyuk et al. (2007) demonstrated that octreotide, a somatostatin analog, reduced cAMP levels in cholangiocytes, which suppressed liver cyst growth and fibrosis [154,155]. In polycystic kidney disease, targeting ciliary dysfunction through cAMP modulation, growth factor inhibition, microRNA-17 inhibition, and mTOR inhibition has demonstrated therapeutic potential [156]. Increased cAMP levels and abnormal vasopressin receptor (V2R) signaling lead to cyst expansion. Tolvaptan, a V2R antagonist, effectively reduces cAMP levels and slows disease progression, although it has hepatotoxic limitations [157,158].

Therapeutic strategies targeting the mTOR pathway in polycystic kidney disease (PKD) have produced inconsistent results. Deletion of the PKD1 or PKD2 gene increases cilium length and activates the mTOR pathway [159]. However, mTOR inhibitors like rapamycin and everolimus have demonstrated limited clinical success. While these treatments resulted in modest reductions in kidney volume, they had minimal effects on renal function and were associated with significant adverse effects [160].

PC function as key sensory organelles, housing G protein-coupled receptors, receptor tyrosine kinases, and ion channels, and their localization is controlled by a diffusion barrier in the transition zone, making them potential therapeutic targets [161,162]. Their structure dynamically regulates cell proliferation and differentiation, and ciliogenesis plays a crucial role in these processes. Modulating ciliogenesis in non-tumor cells may help regulate differentiation under pathological conditions, thereby reducing susceptibility to disease [163].

AURKA inhibits ciliogenesis by phosphorylating histone deacetylase 6 and is overexpressed in various cancers. Targeting AURKA may suppress tumor proliferation by promoting ciliogenesis, with compounds such as iCRT14, bexarotene, alisertib, and tubulin A identified as potential inhibitors [161,164]. Additionally, PC is crucial for Hh signaling, affecting liver fibrosis and regeneration through smoothened translocation [165,166]. Promising therapeutic targets include USP8, the USP8-Trichoplein-AurA pathway, and USP54, which may enhance ciliogenesis and inhibit tumor progression, particularly in colorectal cancer [165,167]. Advanced tools, such as transcriptomics, mass spectrometry, and genome editing, are crucial for elucidating these pathways and expanding therapeutic possibilities.

PC has become a significant therapeutic target in cancer due to its role in critical processes, such as adaptation to hypoxia, resistance to apoptosis, autophagy, angiogenesis, metabolic reprogramming, and migration, all of which contribute to metastasis [153,168]. However, the role of PC in cancer progression is complex and varies across different tumor types. This variation is likely influenced by tissue-specific ciliation patterns and the effects of the tumor microenvironment on cilia dynamics. Several anticancer compounds have been developed to target cilia dynamics. For instance, alisertib inhibits AURKA to prevent ciliary disassembly, while vinblastine disrupts microtubules to reduce ciliation [168]. Additionally, as signaling hubs, primary cilia are targeted by drugs such as Sonidegib [169] and Vismodegib [170], which inhibit Hh signaling pathways.

PC interacts with HIFs and the tumor microenvironment. HIF inhibitors, such as Belzutifan, which specifically target HIF-2α, have demonstrated promising results in cancers related to von Hippel-Lindau syndrome. These inhibitors work by preventing the accumulation of non-hydroxylated HIF-2α under hypoxic conditions. By inhibiting HIF-2α, they reduce angiogenesis, cell proliferation, and tumor growth, highlighting the potential of targeting pathways associated with low-oxygen levels [36]. In addition, primary cilia (PC) play a crucial role in regulating immune responses and vascular permeability, which influences both metastasis and the outcomes of cancer therapies. When primary cilia are absent, the expression levels of ZO-1, a protein essential for tight junctions, are decreased. This reduction results in disorganization of intercellular junctions and an increase in endothelial permeability. Furthermore, many anticancer drugs unintentionally affect the structure and function of cilia. This highlights the importance of evaluating the impact of these drugs on ciliary dynamics in order to optimize cancer treatments and minimize resistance mechanisms [171].

## 5. Conclusions

In summary, these findings enhance our understanding of the effects of hypoxia on cilia at the molecular level, providing new avenues for research and valuable insights into ciliary dysfunction associated with various diseases. Although few studies have focused specifically on the liver, we believe that this area has significant potential for exploration. While existing research suggests a connection between hypoxia, the HIF pathway, and other molecular pathways that regulate cilia, the evidence is fragmented and occasionally contradictory. This highlights the need for further investigation to clarify these complex interactions.

The potential of primary cilia as a key focus for research on liver pathologies and complications following transplantation is promising, particularly because hypoxia and cilia-associated pathways, such as AURKA, HIFs, and mTOR, play critical roles. Improving our understanding of the relationship between cilia dynamics and hypoxic signaling could lead to innovative therapeutic strategies to alleviate liver dysfunction and improve transplant outcomes.

## Figures and Tables

**Table 1:** Genes Associated with ciliopathy and liver dysfunction.

Gene	Function	Disease	Impact of Pathogenic Variants	Refs.
PKD1 and PKD2	Encode polycystin-1 and polycystin-2, which are involved in calcium signaling and maintaining the structure of primary cilia	Polycystic Liver Disease (PLD) and Autosomal Dominant Polycystic Kidney Disease (ADPKD)	Cyst formation in the liver and kidneys, causing cystic enlargement and disruption of organ function	[65,73,74]
PKHD1	Encodes fibrocystin/polyductin, important for maintaining the architecture of the bile ducts and renal tubules	Autosomal Recessive Polycystic Kidney Disease (ARPKD), Caroli Syndrome and Congenital Hepatic Fibrosis (CHF)	Ductal plate malformations formation, leading to fibrosis and cyst formation in the liver and kidneys	[59,73,75]
DCDC2	Encodes doublecortin domain-containing protein 2, involved in microtubule organization and ciliary function	Neonatal Sclerosing Cholangitis and Biliary Atresia	Disruption in bile duct development and function, leading to cholestasis and liver fibrosis	[77,78]
IFT88	Encodes a protein essential for intraflagellar transport, crucial for cilia formation and maintenance	Bardet-Biedl Syndrome (BBS) and Hepatic Fibrocystic Disease	Defective cilia lead to multi-organ fibrosis, including liver involvement, and other systemic manifestations	[73,74]
ALMS1	Encodes a protein involved in ciliary function and cellular signaling pathways	Alström Syndrome	Results in steatosis, with progressive liver fibrosis, along with retinal degeneration, cardiomyopathy, and other systemic features	[79,80]
NEK8	Encodes a serine/threonine kinase involved in ciliary function and cell cycle regulation	Nephronophthisis and Hepatic Fibrosis	Cystic kidney disease and liver fibrosis, indicating a shared pathogenesis involving ciliary dysfunction	[82,83,84]
ARL13B	Encodes a GTPase required for normal ciliary function and signaling	Joubert Syndrome and Hepatic Fibrosis	Ciliary signaling disruption, leading to cerebellar and hepatic fibrosis, and other systemic anomalies	[85]
MKS1	Encodes a protein involved in ciliogenesis and centrosome function	Meckel-Gruber Syndrome and Hepatic Fibrosis	Lethal multi-organ fibrosis, including hepatic and renal cysts, and other developmental anomalies	[86,87,88]
mTORC1	Influence the process of ciliogenesis by regulating the synthesis of proteins and lipids required for cilia assembly. It coordinates the cellular growth signals that are necessary for the formation of the ciliary membrane and axoneme	Nonalcoholic fatty liver disease (NAFLD) and nonalcoholic steatohepatitis (NASH)	Alterations in nutrient sensing, autophagy regulation and cellular stress response affecting ciliary function	[95]
MAN1A2	Involved in the processing of N-linked oligosaccharides during glycoprotein biosynthesis	Biliary Atresia	Knockdown of MAN1A2 results in poor biliary network formation and ciliary dysgenesis	[92]
KIF3B	Part of the kinesin-2 motor protein complex, which is essential for the anterograde transport of molecular cargoes along microtubules in cilia		Variants in KIF3B can impair ciliary assembly and maintenance, potentially leading to defects in ciliary signaling pathways	[93]
TTC17	Involved in the organization of ciliary and centrosomal structures. It plays a role in the assembly and stability of ciliary axonemes		Defects in TTC17 can lead to ciliary dysfunction and impaired signaling	[93]
PCNT	Encodes a protein that is a key component of the centrosome and is involved in microtubule organization. It plays a critical role in the formation and function of primary cilia by anchoring and stabilizing the microtubules at the base of the cilium		Variants in PCNT can cause structural and functional defects in cilia, leading to various ciliopathies	[93]
PKD1L1	Encodes a protein that forms part of a ciliary calcium channel complex with PKD2L1. This complex is involved in mechanosensation and signal transduction within cilia		Variants in PKD1L1 have been found in patients with Biliary Atresia Splenic Malformation (BASM) syndrome, indicating a link between ciliary dysfunction and BA. The disruption of PKD1L1 can affect hepatobiliary development	[91,96,97]
Planar polarity genes	Essential for positioning cells in 3D networks to establish the proper morphogenesis, structure, and function of organs during embryonic development		BA is closely linked to polygenic susceptibility involving 102 genes related to ciliogenesis and planar polarity effectors. Functional data point to issues in ciliary development, abnormal biliary epithelial cell formation, and disrupted vasculogenesis	[98]

## Data Availability

No new data were created for this manuscript.

## Abbreviations

The following abbreviations are used in this manuscript:

ADPLD	Autosomal dominant polycystic liver disease
AURKA	Aurora kinase A
BA	Biliary atresia
BASM	Biliary atresia-associated splenic malformation
CABA	Cardiac-associated biliary atresia
CD	Caroli Disease
CDG	Congenital Disorder of Glycosylation
CHF	Congenital Hepatic Fibrosis
DPM	Ductal plate malformation
DR	Ductular reaction
ER	Endoplasmic reticulum
FGF-2	Fibroblast growth factor-2
GWAS	Genome-wide association studies
HIF	Hypoxia-inducible factors
HIF-3α	Hypoxia-inducible factor 3 alpha
HRE	Hypoxia-responsive element
IC	Ischemic cholangiopathy
IFT	Intraflagellar transport
IFT-A	IFT complex A
IFT-B	IFT complex B
KIF3B	Kinesin-like protein KIF3B
LTx	Liver transplantation
MAN1A2	Mannosidase alpha class 1A member 2
MSC	Mesenchymal stem cells
PC	Primary cilia
PCNT	Pericentrin
PLD	Polycystic Liver Disease
pVHL	Von Hippel-Lindau tumor suppressor protein
PVP	Peribiliary vascular plexus
TRPV4	Transient receptor potential cation subfamily V member 4
TTC17	Tetratricopeptide repeat domain 17
USP8	Ubiquitin-specific protease 8
V2R	Vasopressin receptor
VDAC1-ΔC-	Voltage-gated anion channel
VHL	Von Hippel-Lindau
